# Commentary: Guselkumab binding to CD64^+^ IL-23–producing myeloid cells enhances potency for neutralizing IL-23 signaling

**DOI:** 10.3389/fimmu.2025.1604337

**Published:** 2025-05-20

**Authors:** Giulia Valdiserra, Clelia Di Salvo, Matteo Fornai, Luca Antonioli

**Affiliations:** Department of Clinical and Experimental Medicine, University of Pisa, Pisa, Italy

**Keywords:** IL-23, IL-23p19 subunit inhibitors, guselkumab, CD64, pain, immune-mediated inflammatory diseases

Immune-mediated inflammatory diseases (IMIDs) are a heterogeneous group of disorders—including gastroenteric, rheumatologic, dermatologic, neurologic, and systemic autoimmune conditions—characterized by chronic inflammation and intermittent flares (1). While a wide range of pro-inflammatory mediators are involved in the pathophysiology of IMIDs, in certain subsets such as Crohn’s disease, psoriasis, and psoriatic arthritis, specific cytokines including tumor necrosis factor (TNF) and interleukin-23 (IL-23) have emerged as key drivers of inflammation (1). In these conditions, TNF and IL-23 can act as “master regulators” by orchestrating immune cell activation and sustaining inflammatory responses, thus representing important therapeutic targets. However, this centrality is not consistent across all IMIDs; in other diseases such as multiple sclerosis or systemic lupus erythematosus, these pathways may be less prominent or therapeutically unsuitable (1).

Over the years, IL-23, a member of the IL-12 cytokine family, has emerged as a hierarchically dominant regulatory cytokine capable of reprogramming immune cells to induce and maintain a highly proinflammatory state (2). It acts as a “master switch” in the development and persistence of certain IMIDs. IL-23 profoundly affects both innate immune cells (e.g., macrophages, dendritic cells, ILC3s, and neutrophils) and adaptive immunity by promoting the differentiation of naïve T cells into the T_H17_ phenotype (3). Notably, a subset of mononuclear phagocytes, such as macrophages, dendritic cells or CD64brightCD163^−^CD14brightCD1c^−^CD1a^–^ inflammatory monocyte-like cells, are key sources of IL-23 in response to various physiological and pathological stimuli (3).

Structurally, IL-23 is a heterodimeric cytokine composed of the proteins p19 and p40. While p19 is the unique subunit of IL-23, p40 is the common subunit shared with IL-12 (3). In recent years, the monoclonal antibodies risankizumab, guselkumab, mirikizumab, and tildrakizumab, which selectively target the p19 subunit, have been introduced into clinical practice for the management of several IMIDs (2). The efficacy of these selective IL-23 blockers has been well-established in conditions such as psoriasis, psoriatic arthritis, and IBDs (Crohn’s disease and ulcerative colitis) (4).

Although all anti-IL 23 antibodies are endowed with a marked inhibitory effect on IL-23, with variable K_D_ values, they displayed some structural differences (5). Guselkumab is a fully human IgG1 monoclonal antibody with a native Fc region, while risankizumab and mirikizumab are humanized IgG1 and IgG4 variant monoclonal antibody, respectively, with a mutated Fc region (5).

In this commentary, we focus on the recent original research paper by Sachen et al. (4) highlighting a pharmacodynamic peculiarity of guselkumab. Due to its native Fc domain, guselkumab binds to the CD64 protein expressed on IL-23– producing myeloid cells, leading to internalization and trafficking of IL-23 to endolysosomal compartments. This seems to enhance its potency in neutralizing IL-23 signaling (4).

CD64, also known as high-affinity IgG receptor (FcγRI), is constitutively expressed on most myeloid cells, including macrophages, monocytes, and dendritic cells. It can also be induced by cytokines on neutrophils, mast cells, and eosinophils (6). Proinflammatory cytokines TNF or IFN-γ upregulated CD64 expression on dendritic cells and monocytes (6).

CD64 binds to the Fc portion of immunoglobulin IgG1, IgG3 and IgG4 enabling immune cells to recognize and interact with immune complexes, trigger the release of proinflammatory cytokines, promote phagocytosis, and amplify the immune response (6).

**Figure 1:**
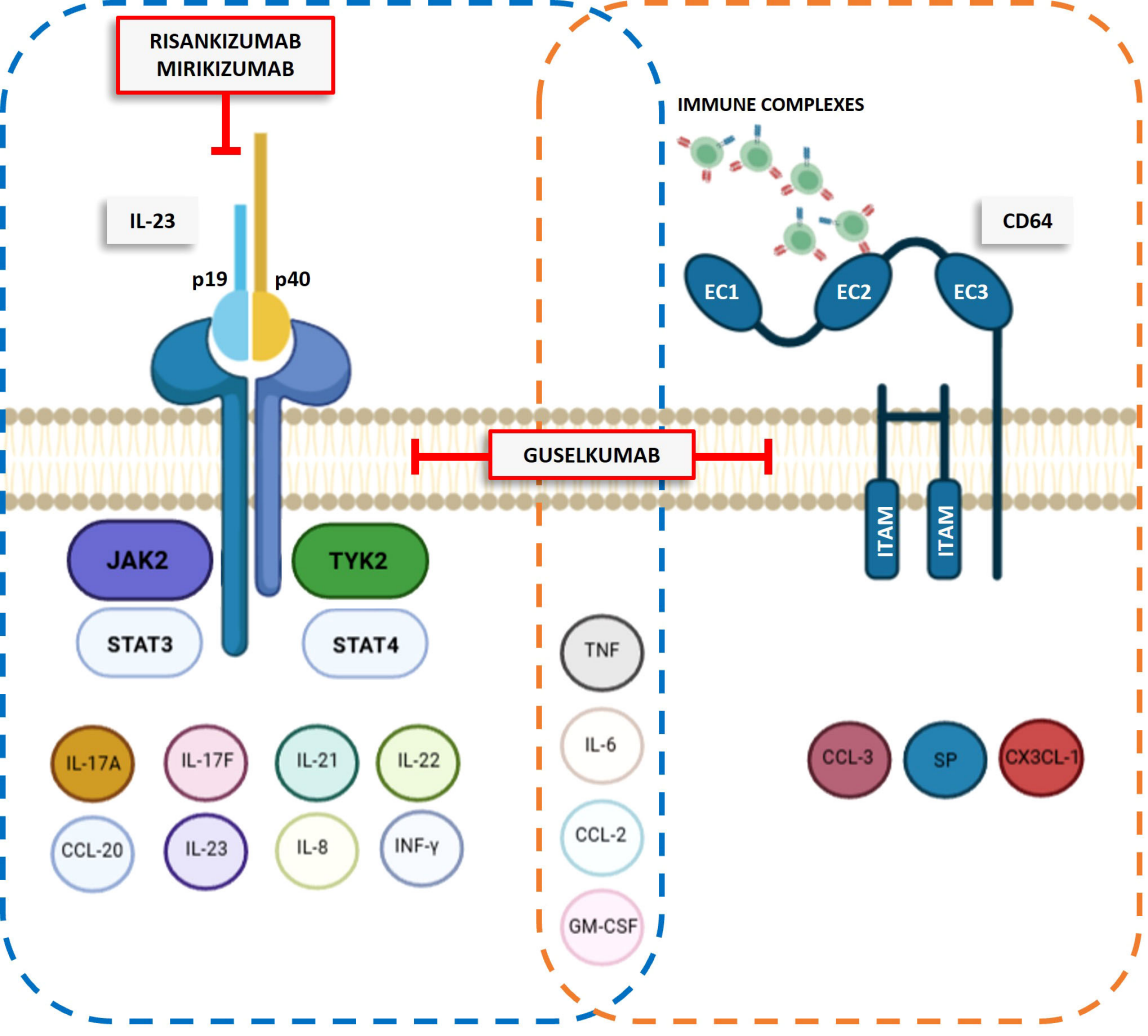
IL-23, through the JAK/STAT pathway, promotes the release of various cytokines, including IL-17A, IL-17F, IL-21, IL-22, CCL-20, IL-23, IL-8, INF-γ, TNF, IL-6, CCL-2, and GM-CSF. CD64 activation by immune complexes triggers the release of CCL-3, substance P (SP), CX3CL-1, TNF, IL-6, CCL-2, and GM-CSF. Anti-IL-23 antibodies, such as risankizumab, mirikizumab, and guselkumab, inhibit IL-23 signaling by targeting its p19 subunit. Additionally, guselkumab reduces CD64 expression, thereby weakening the downstream signaling associated with this protein.

Importantly, Sachen et al. (4) demonstrated that guselkumab despite its binding with CD64 did not induce cytokine secretion from myeloid cells stimulated with TLR ligands. It is worth noting that CD64 activation also requires stimulation of ITAM region (see **Figure 1**; a short peptide sequences that play a crucial role in transmitting signals inside the cell following CD64 activation) to induce the release of pro-inflammatory cytokines and reactive oxygen species (6). It is plausible that guselkumab merely binds the EC2 subunit of CD64 without activating the ITAM signaling pathway.

When evaluating the functional consequences of CD64 binding on IL-23–targeting monoclonal antibodies (mAbs), it was observed that guselkumab and risankizumab showed similar potency in monoculture cellular assays with exogenous addition of IL-23 (4). However, in a co-culture system involving IL-23–producing CD64^+^ THP-1 cells and an IL-23–responsive reporter cell line, guselkumab demonstrated approximately 10-fold greater potency for IL-23 signaling inhibition, in an Fc-dependent manner, compared to risankizumab (4). The authors ascribed this enhanced potency to more effective neutralization of locally produced IL-23 via CD64 binding and Fc domain interaction (4).

Further *in vitro* studies revealed that guselkumab–IL-23 complexes are internalized into lysosomes within CD64^+^ macrophages. Moreover, guselkumab can be internalized independently of IL-23 binding, without compromising its IL-23 neutralization capacity (4).

The observation that guselkumab, in addition to efficiently inhibiting IL-23 activity, also downregulates CD64 expression, opens new perspectives regarding its potential impact on macrophage function and broader immune modulation.

Recent findings have also highlighted CD64 expression on non-immune cells. Notably, CD64 has been localized on primary sensory neurons (7) and dorsal root ganglia (DRG neurons) (6), especially under inflammatory conditions such as neuropathic pain, nerve injury, or chronic inflammation. Kolter et al. (8) described a population of CD64^+^ macrophages associated with sensory nerves and enteric nerves. This suggests a possible role for CD64 in immune–neural interactions, potentially contributing to pain signaling and neuroinflammation. Preclinical evidence shows that IgG immune complexes can activate joint sensory neurons in a FcγRI-dependent manner, triggering acute joint pain in the absence of overt inflammation (9).

Although these findings offer compelling *in vitro* insights, future studies using relevant *in vivo* models are needed to determine whether differences in Fcγ receptor engagement, such as preferential CD64 binding, influence the therapeutic efficacy, pharmacokinetics, or tissue distribution of IL-23 inhibitors. This is particularly relevant in the absence of head-to-head clinical trials.

If validated by further studies, the unique ability of guselkumab to disrupt CD64-mediated pathways could pave the way for novel clinical applications. These may include diseases driven by CD64^+^ cells—such as vasculitis, granulomatosis, or sarcoidosis—as well as immune-mediated disorders associated with pain states that are poorly controlled by existing therapies, including those not primarily driven by inflammation (e.g., visceral pain).
